# Trigger Criteria for Advance-Care Planning in ACHD: The Time Is Now

**DOI:** 10.1016/j.cjcpc.2025.10.007

**Published:** 2025-10-31

**Authors:** Michelle Keir, Jennifer Ann Sullivan

**Affiliations:** Southern Alberta Adult Congenital Heart Clinic, Libin Cardiovascular Institute, University of Calgary, Calgary, Alberta, Canada

**Keywords:** adult congenital heart disease, advance-care planning, palliative care, fontan

The care of congenital heart disease (CHD) has radically transformed over the past century.^1^ Early paediatric cardiologists often had little to offer parents but a grim prognosis and condolences.^2^ Today, adult CHD practitioners can confidently proclaim that our goal is to help our patients reach a normal life expectancy.^3^ For some conditions, though, such as hypoplastic left heart syndrome, a death in early childhood has only been postponed to early adulthood or midlife.^4^^,^^5^ This means that, although their contemporaries are planning families and careers, many young adults with CHD must also confront their own mortality and make care decisions usually faced by much older people.^6^ Complex decisions regarding death and dying, that were hitherto faced by parents, now fall to adults with CHD. It has become the job of their adult CHD care team to guide them through these tough decisions.

Possner et al.^7^ confront a crucial problem facing adult CHD providers; when is the right time to discuss prognosis, life expectancy, and disease trajectory with teenagers and young adults? How do we balance the need for optimism and hope with realistic expectations? Patients with CHD have repeatedly signalled that these conversations are important to their longitudinal care.^8^^,^^9^ Despite the importance of these conversations to both patients and providers, we know that they are not happening with regularity.^10^^,^^11^ An important first step in improving care for patients with CHD is the establishment of trigger criteria, or clinical red flag scenarios, that should prompt a discussion about advance-care planning (ACP).

The systematic review of the current literature for trigger criteria in ACP in the CHD population, presented by Possner et al., provides important insights.^7^ Much of the reviewed literature, nearly half, has been published in the past 5 years. This signals the importance of this problem in contemporary CHD practice. It also displays the shift in CHD research from prolonging life expectancy to improving quality of life.^12^ Possner et al. present the first quality review of the available evidence. They describe moderate-quality research with significant heterogeneity. The authors identify that much of the research has been conducted in high-income, English-speaking countries, leaving the unmet opportunity of assessing values and preferences in diverse cultural and economic environments.

The list of trigger criteria identified in the literature covers nearly every clinical scenario; transition, employment considerations, family planning, preprocedure or surgery, and transplant or mechanical circulatory support discussions. It can be argued that this compilation suggests that ACP should be a standard part of routine clinical care for patients with CHD and their families. In fact, normalizing ACP conversations and decisions is important for all adults, regardless of health status. There are robust conversation guides and decision templates available to all Canadians, which could be used by CHD practitioners.^13^

This presents the challenge of feasibility. The landscape of adult CHD care is resource-limited, even in high-income countries. The change in mortality, complexity, and care needs of patients with CHD means that there is a burgeoning number of patients with CHD in midlife with a dearth of trained adult CHD cardiologists.^14^ Even in a shared-care model, the anatomic and clinical heterogeneity of the adult CHD population means general cardiologists often feel ill-equipped to have these nuanced conversations about life expectancy and disease trajectory.^15^ Heart failure can present more subtly in patients with CHD than in the traditional, older heart failure population, and the signs of advancing disease can be missed.^16^ In addition, outdated compensation models favour procedures over counselling in adult cardiology.^17^ The research by Possner et al.^7^ reinforces the need to expand the adult CHD workforce and reconsider compensation models to meet the increasing demands of caring for this complex population.

An unanswered quandary is how to support patients and families where neurocognitive deficits are present. We know that, although most adults with CHD have a normal intelligence, many deal with subtle executive dysfunction due to foetal and neonatal cyanosis.^18^ The lack of standardized paradigms for assessing these cumulative neurocognitive differences is an additional barrier to supportive decision-making in the CHD population.^19^ Also, many patients with CHD deal with concomitant mental health conditions (anxiety, depression, and post-traumatic stress disorder) that can increase the complexity of ACP discussions.^20^ Adult CHD clinical providers require continuing education opportunities to support them in having these nuanced patient-specific conversations.

In conclusion, there is no wrong time to address life expectancy and initiate ACP discussions with patients with CHD. The challenge is finding the time and expertise to do so.

## Funding Sources

The authors have no funding sources to declare.

## Disclosures

The authors have no conflicts of interest to disclose.
